# Extensive Dural Metastasis as Initial Presentation of Metastatic Prostate Cancer: A Case Report With Durable Disease Control Following Multimodal Treatment

**DOI:** 10.1155/criu/1896431

**Published:** 2026-11-04

**Authors:** David Wasilewski, Jörg Felsberg, Theodoros Tzamalis, Sajjad Muhammad

**Affiliations:** ^1^ Klinik für Neurochirurgie Universitätsklinikum Düsseldorf Moorenstr Düsseldorf Germany; ^2^ German Cancer Consortium (DKTK) Partner Site Düsseldorf and German Cancer Research Center (DKFZ) Heidelberg Germany dkfz.de; ^3^ Institut für Neuropathologie Universitätsklinikum Düsseldorf Moorenstr Düsseldorf Germany; ^4^ Klinik für Hämatologie und Onkologie Marienhospital Düsseldorf Rochusstr Düsseldorf Germany

**Keywords:** case report, central nervous system metastasis, dural metastasis, pachymeningeal metastasis, prostate cancer

## Abstract

Central nervous system involvement in prostate cancer is rare, particularly in the form of dural or pachymeningeal metastases. We report the case of a 72‐year‐old man presenting with visual disturbances without hemianopsia, and right‐sided headache since several weeks caused by an extensive right temporal dural‐based lesion with intracranial extension. A minimally invasive frontotemporal burr‐hole biopsy was performed for histopathological clarification. Histological and immunohistochemical analyses confirmed metastatic prostatic adenocarcinoma with expression of cytokeratin 8, NKX3.1, PSA, and PSMA. Subsequent systemic staging demonstrated metastatic castration‐sensitive prostate cancer with pulmonary, lymphatic, and extensive osseous metastases. The patient underwent focal radiotherapy (36 Gy in 12 fractions) to the dural lesion and systemic treatment with androgen deprivation therapy and enzalutamide. Due to dizziness and sweating, enzalutamide dosage was reduced from 160 to 80 mg/day with maintained therapeutic response. At approximately 15 months of follow‐up, the patient remained neurologically stable with durable local disease control on cranial MRI, marked biochemical response (PSA decline from 177 to 0.6 ng/mL), and no evidence of systemic progression. This case highlights the importance of considering metastatic prostate cancer in the differential diagnosis of dural‐based intracranial lesions in elderly men and demonstrates that individualized multimodal treatment can achieve durable disease control even in rare metastatic presentations.

## 1. Introduction

Prostate cancer most commonly metastasizes to bone, lymph nodes, and visceral organs. Central nervous system (CNS) involvement is distinctly uncommon, with contemporary series reporting clinically detected brain metastases in approximately 1%–2% of patients with advanced prostate cancer [1, 2]. Among these rare manifestations, dural or pachymeningeal metastases represent an even less frequent subset and may radiologically mimic meningioma or other dural‐based lesions [1–3].

Most CNS metastases from prostate cancer occur metachronously several years after the initial diagnosis and are typically associated with advanced metastatic castration‐resistant prostate cancer (mCRPC) [1, 3, 4]. In contrast, synchronous CNS involvement at the time of prostate cancer diagnosis is exceptionally rare and has been described primarily in isolated case reports and small retrospective series [1, 5–8]. Reported presentations range from solitary intraparenchymal metastases to extensive dural or skull‐base lesions initially mistaken for primary intracranial tumors [5–9].

Here, we report a case of newly diagnosed metastatic castration‐sensitive prostate cancer (mCSPC) presenting with an extensive right temporal dural metastasis as the initial manifestation of disease. Histopathological diagnosis was established through minimally invasive neurosurgical biopsy, followed by focal radiotherapy and systemic treatment with androgen deprivation therapy and enzalutamide. This multimodal approach resulted in durable local and systemic disease control over 15 months of follow‐up. This case report was prepared in accordance with the CARE reporting guideline. The completed CARE checklist is provided as Supporting Information (File S1: CARE_checklist).

## 2. Case Report

### 2.1. Initial Findings

A 72‐year‐old male with hypertension and carotid artery disease presented in October 2023 with visual disturbances and intermittent right‐sided headaches for approximately 4 weeks (Table 1). He denied other neurological symptoms, including limb weakness, speech difficulties, or seizures. On neurological examination, the patient was awake and fully oriented, without focal motor or sensory deficits, aphasia, cranial nerve palsy, or clinical signs of seizure activity.

**Table 1:** Timeline.

Date	Event
October 2023	Presentation with headaches and visual disturbances
October 2023	Cranial MRI demonstrating right temporal dural‐based lesion
October 2023	Minimally invasive frontotemporal burr‐hole biopsy
December 2023	CT staging demonstrating disseminated metastatic disease
January 2024	Bone scintigraphy and initiation of androgen deprivation therapy
February 2024	Initiation of enzalutamide (160 mg/day)
February–March 2024	Radiotherapy to dural lesion (36 Gy in 12 fractions)
April 2024	Enzalutamide dose reduction to 80 mg/day
July 2024	PSA decline to 0.6 ng/mL
January 2025	Stable intracranial and systemic disease on follow‐up imaging

There was no family history of malignancy. An outpatient cranial MRI performed at the beginning of October revealed a large dural‐based lesion in the right temporal region, accompanied by marked surrounding perifocal edema (Figure 1 A–D). Radiologically, the lesion was considered suspicious for metastatic disease or atypical meningioma. Due to the extent of dural and intracranial involvement, an intracranial tissue biopsy was indicated to obtain tissue for diagnostic evaluation. Given the extensive dural involvement and the unclear underlying diagnosis, a minimally invasive right frontotemporal burr‐hole biopsy with dural opening and tissue sampling was performed under general anesthesia to establish histopathological diagnosis while minimizing surgical morbidity. Gross total resection was not pursued because of the extensive infiltrative dural involvement and lack of significant mass effect requiring decompression.

**Figure 1:**
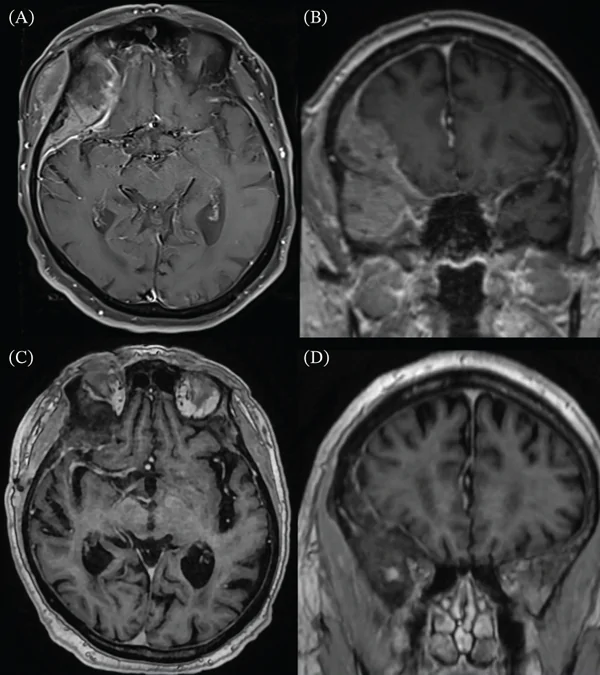
(A–D) Cranial MRI at diagnosis and follow‐up. Pretreatment contrast‐enhanced T1‐weighted cranial MRI obtained in October 2023. (A) Axial and (B) coronal sequences demonstrate an extensive right temporal dural‐based enhancing lesion with pachymeningeal thickening and surrounding vasogenic edema. Follow‐up MRI obtained in January 2025. (C) Axial and (D) coronal contrast‐enhanced sequences show durable local disease control without evidence of recurrent dural metastatic disease.

Systemic staging with contrast‐enhanced computed tomography and bone scintigraphy revealed disseminated metastatic disease, including retroperitoneal lymphadenopathy, pulmonary nodules, and extensive osteoblastic skeletal metastases involving the spine, pelvis, and skull base (Figures 2A–C; 3A,B; and 4A,B).

**Figure 2:**
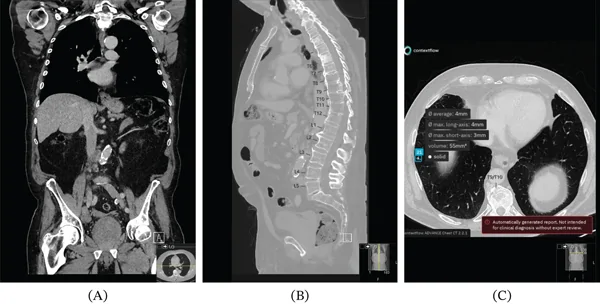
(A–C) Baseline systemic staging CT from December 2023. (A) Coronal contrast‐enhanced CT demonstrates enlarged retroperitoneal and para‐aortic lymph nodes consistent with lymphatic metastatic disease. (B) Sagittal bone‐window CT reveals multiple osteoblastic vertebral lesions involving the thoracic spine. (C) Axial chest CT demonstrates a small solid pulmonary nodule in the right lower lobe, suspicious for pulmonary metastatic involvement.

**Figure 3:**
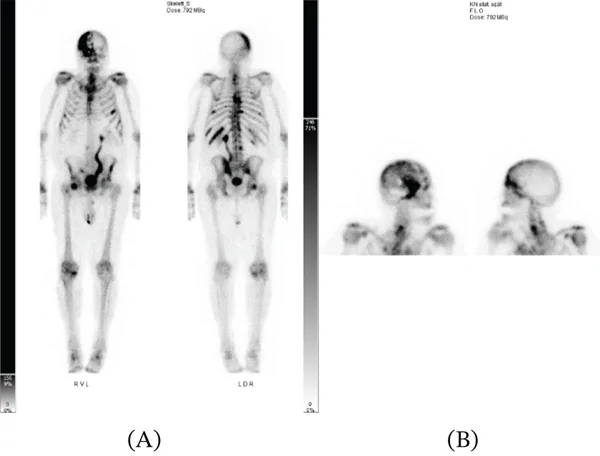
(A,B) Baseline bone scintigraphy from January 2024. (A) Whole‐body Tc‐99 m DPD bone scintigraphy demonstrates extensive disseminated skeletal metastatic disease involving the axial skeleton, pelvis, and proximal femora. (B) Focused skull images demonstrate increased radiotracer uptake involving the right temporal skull base region, consistent with osseous and adjacent dural metastatic involvement.

**Figure 4:**
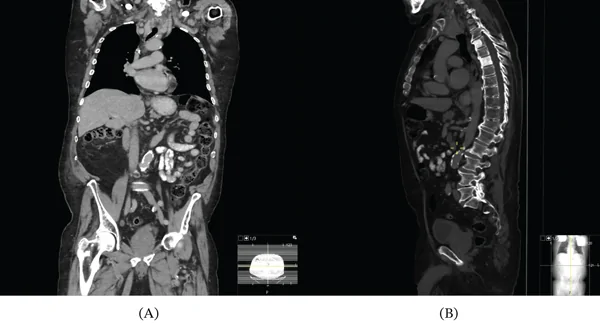
(A,B) Follow‐up systemic staging CT from January 2025. (A) Coronal contrast‐enhanced CT demonstrates stable retroperitoneal lymphadenopathy and regression of previously described pulmonary metastatic lesions without evidence of progressive visceral disease. (B) Sagittal CT reconstruction demonstrates stable osteoblastic vertebral metastases without radiographic progression.

### 2.2. Histopathological Findings

Microscopic examination revealed an infiltrating epithelial neoplasm composed of atypical tumor cells arranged in solid and glandular structures within fibrous dural tissue. Tumor cells demonstrated enlarged hyperchromatic nuclei and eosinophilic cytoplasm.

Immunohistochemical analysis showed diffuse positivity for cytokeratin 8, prostate‐specific antigen (PSA), NKX3.1, and PSMA, supporting the diagnosis of metastatic prostatic adenocarcinoma (Figure 5A–D). The immunophenotypic profile, together with the clinical and radiological findings, confirmed the diagnosis of metastatic prostate cancer involving the dura.

**Figure 5:**
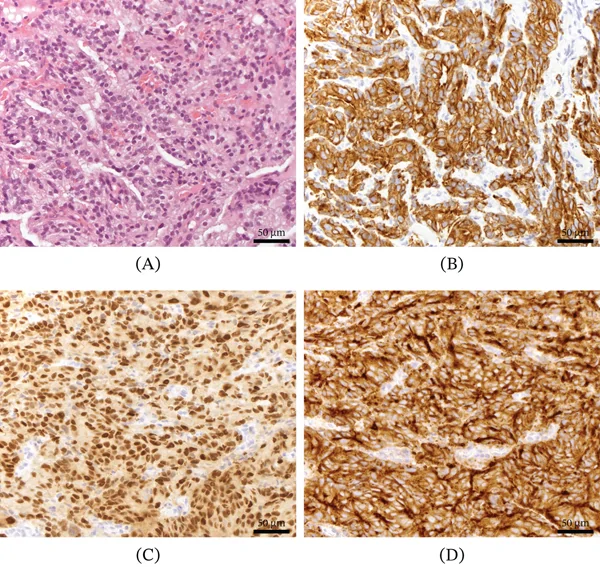
(A–D) Histopathological and immunohistochemical findings. (A) Hematoxylin and eosin staining demonstrates infiltrating epithelial tumor cells within fibrous dural tissue. (B) Immunohistochemistry shows diffuse positivity for cytokeratin 8. (C) Strong nuclear expression of NKX3.1 confirms prostatic lineage. (D) Strong membranous PSMA expression further supports metastatic prostatic adenocarcinoma. Scale bars: 50 *μ*m.

### 2.3. Adjuvant Treatment and Follow‐Up

Baseline PSA level was 177 ng/mL. The patient was diagnosed with newly diagnosed mCSPC. Systemic therapy with androgen deprivation therapy (Trenantone 11.25 mg) was initiated in January 2024, followed by enzalutamide (160 mg/day) in February 2024. In addition, focal external beam radiotherapy to the dural lesion was administered with a total dose of 36 Gy in 12 fractions.

During treatment, the patient experienced dizziness and sweating attributable to enzalutamide therapy, necessitating dose reduction to 80 mg/day in April 2024. Nevertheless, biochemical response remained excellent, with PSA declining to 0.6 ng/mL by July 2024.

Serial follow‐up imaging demonstrated durable local disease control with shrinkage of the dural lesion and lack of recurrence of the dural lesion (Figure 1C,D), partial remission of pulmonary and nodal metastases, and stable osseous metastatic disease (Figure 4A,B). At the most recent follow‐up in January 2025, approximately 15 months after diagnosis, the patient remained neurologically stable without new deficits or seizures.

## 3. Discussion

Brain metastases from prostate cancer are rare, with contemporary series reporting incidences of approximately 1%–2% [1, 2]. Dural or pachymeningeal metastases represent an even less common manifestation and are typically encountered in patients with advanced disease, extensive skeletal involvement, and castration‐resistant disease [1–4]. The present case is unusual because the dural metastasis was identified synchronously with the primary diagnosis of mCSPC, making it the initial clinical manifestation of the underlying malignancy.

The distinction between parenchymal brain metastases and dural metastases is clinically relevant. Although parenchymal lesions are generally believed to arise through hematogenous dissemination, dural metastases may additionally result from direct extension of calvarial or skull‐base metastases into the dura [1, 3, 4]. In our patient, the extensive skull‐base involvement demonstrated on bone scintigraphy supports this mechanism. Radiologically, dural metastases often resemble meningiomas because they appear as extra‐axial enhancing lesions with broad dural attachment and surrounding vasogenic edema [6–9]. Consequently, histopathological confirmation remains essential, particularly in patients without a known history of malignancy.

Histopathological confirmation was a key aspect of the present case. The lesion was initially considered suspicious for either metastatic disease or atypical meningioma. Minimally invasive burr‐hole biopsy enabled definitive diagnosis while avoiding the morbidity associated with extensive resection of an infiltrative dural lesion in the setting of disseminated systemic disease. Immunohistochemistry demonstrated strong expression of PSA, PSMA, NKX3.1, and cytokeratin 8, providing compelling evidence of prostatic origin. Among these markers, NKX3.1 has emerged as one of the most sensitive and specific markers for metastatic prostatic adenocarcinoma and is particularly valuable in unusual metastatic presentations.

Another noteworthy aspect of this case is the favorable clinical course despite extensive metastatic burden. Historically, prognosis following diagnosis of CNS metastases from prostate cancer has been poor, with median overall survival ranging from approximately 4–6 months in most retrospective series [1, 3, 4]. Patients with dural metastases generally have even shorter survival than those with isolated intraparenchymal lesions [1]. However, recent reports suggest that selected patients with synchronous CNS involvement may achieve prolonged survival when treated using multimodal strategies [5, 10].

Following histopathological diagnosis, our patient received focal radiotherapy and systemic treatment with androgen deprivation therapy and enzalutamide. Treatment intensification with androgen receptor pathway inhibitors has become a standard component of care for mCSPC following the ENZAMET and ARCHES trials, which demonstrated significant improvements in overall survival and progression‐free survival compared with androgen deprivation therapy alone [11, 12]. Although evidence regarding the intracranial efficacy of enzalutamide remains limited, the drug is known to cross the blood–brain barrier and may achieve clinically relevant CNS concentrations. Nevertheless, direct conclusions regarding intracranial activity cannot be drawn from a single case, and the favorable outcome observed in our patient most likely reflects the combined effects of local radiotherapy and effective systemic disease control.

At approximately 15 months of follow‐up, the patient demonstrated durable local control of the dural lesion, marked biochemical response with PSA reduction from 177 to 0.6 ng/mL, regression of pulmonary and nodal metastases, and stable osseous disease. Although isolated case reports cannot establish treatment recommendations, this outcome supports the concept that aggressive multidisciplinary management may achieve meaningful disease control in selected patients with synchronous CNS involvement.

Several reports have described dural or skull‐base metastases as the first manifestation of prostate cancer. Franco et al. reported sphenoid lesions initially interpreted as meningiomas that were ultimately diagnosed as metastatic prostate adenocarcinoma following tissue acquisition [7]. Similarly, Rahmathulla et al. described a skull‐base lesion radiologically indistinguishable from meningioma, with the diagnosis established only after surgical resection and histopathological evaluation [8]. Additional reports have emphasized that dural metastatic lesions can closely mimic primary intracranial tumors and therefore require a high index of clinical suspicion [6, 9]. More recently, Parillo et al. described nonenhancing prostate cancer brain metastases identified with delayed postcontrast MRI, further illustrating the radiological heterogeneity of CNS involvement in prostate cancer despite a different imaging phenotype from the dural‐based enhancing lesion observed in our patient [13]. Collectively, these observations highlight the importance of including metastatic prostate cancer in the differential diagnosis of unusual intracranial lesions in elderly men, particularly in the presence of osteoblastic skeletal lesions or elevated serum PSA levels.

Strengths of this case include histopathological confirmation with a prostate‐specific immunohistochemical profile, comprehensive multimodal staging, and longitudinal clinical and radiological follow‐up. Limitations are inherent to the single‐patient design. In particular, the relative contributions of focal radiotherapy and systemic treatment to intracranial disease control cannot be separated, and the findings cannot be generalized to broader patient populations.

## 4. Conclusion

This case describes an exceptionally rare presentation of mCSPC with extensive dural and pachymeningeal involvement as the initial manifestation of disease. Histopathological confirmation through minimally invasive neurosurgical biopsy enabled prompt multidisciplinary treatment.

Combined radiotherapy, androgen deprivation therapy, and enzalutamide resulted in durable local and systemic disease control over approximately 15 months of follow‐up. Although conclusions from a single case remain limited, this report emphasizes the importance of considering metastatic prostate cancer in the differential diagnosis of dural‐based intracranial lesions in elderly men and supports the potential value of individualized multimodal management strategies.

## Author Contributions

David Wasilewski: conceptualization, investigation, data curation, visualization, writing—original draft, and project administration. Jörg Felsberg: investigation, neuropathological evaluation, resources, visualization, and review. Theodoros Tzamalis: clinical data acquisition review. Sajjad Muhammad: conceptualization, supervision, and writing—review and editing.

## Funding

No funding was received for this manuscript.

## Disclosure

All authors have read and approved the final version of the manuscript and agreed to be accountable for the work.

## Consent

Written informed consent was obtained from the patient for publication of this case report and the accompanying clinical, radiological, and histopathological images.

## Conflicts of Interest

The authors declare no conflicts of interest.

## Supporting information


**Supporting Information** Additional supporting information can be found online in the Supporting Information section. File S1: (CARE_checklist): Completed CARE checklist indicating where the recommended case‐reporting items are addressed in the manuscript.

## Data Availability

The data that support the findings of this study are available on request from the corresponding author. The data are not publicly available due to privacy or ethical restrictions.
