# Supplementary material for: Extensive Dural Metastasis as Initial Presentation of Metastatic Prostate Cancer: A Case Report With Durable Disease Control Following Multimodal Treatment
Source: Case Rep Urol. 2026 Nov 4;2026:1896431. doi: 10.1155/criu/1896431 (PMC13736075; doi:10.1155/criu/1896431)
Supplement: Supplementary file 1 [file CRIU-2026-1896431-s001.pdf]

CARE case report guidelines

CARE Checklist of information to include when writing a case report

(cc) BY-NC-ND

Topic	Item	Checklist item description	Reported on Line
Title	1	The diagnosis or intervention of primary focus followed by the words "case report"	Title
Key Words	2	2 to 5 key words that identify diagnoses or interventions in this case report, including "case report"	Keywords
Abstract (no references)	3a	Introduction: What is unique about this case and what does it add to the scientific literature?	Abstract
	3b	Main symptoms and/or important clinical findings	Abstract
	3c	The main diagnoses, therapeutic interventions, and outcomes	Abstract
	3d	Conclusion—What is the main "take-away" lesson(s) from this case?	Abstract
Introduction	4	One or two paragraphs summarizing why this case is unique (**may include references**)	Introduction
Patient Information	5a	De-identified patient specific information.	Initial findings
	5b	Primary concerns and symptoms of the patient.	Initial findings
	5c	Medical, family, and psycho-social history including relevant genetic information	Initial findings
	5d	Relevant past interventions with outcomes	N/A
Clinical Findings	6	Describe significant physical examination (PE) and important clinical findings.	Initial findings
Timeline	7	Historical and current information from this episode of care organized as a timeline	Timeline
Diagnostic Assessment	8a	Diagnostic testing (such as PE, laboratory testing, imaging, surveys).	Case report
	8b	Diagnostic challenges (such as access to testing, financial, or cultural)	N/A
	8c	Diagnosis (including other diagnoses considered)	Case report
	8d	Prognosis (such as staging in oncology) where applicable	Case report
Therapeutic Intervention	9a	Types of therapeutic intervention (such as pharmacologic, surgical, preventive, self-care)	Case report
	9b	Administration of therapeutic intervention (such as dosage, strength, duration)	Adjuvant treatment
	9c	Changes in therapeutic intervention (with rationale)	Adjuvant treatment
Follow-up and Outcomes	10a	Clinician and patient-assessed outcomes (if available)	Treatment / follow-up
	10b	Important follow-up diagnostic and other test results	Treatment / follow-up
	10c	Intervention adherence and tolerability (How was this assessed?)	Adjuvant treatment
	10d	Adverse and unanticipated events	Adjuvant treatment
Discussion	11a	A scientific discussion of the strengths AND limitations associated with this case report	Discussion
	11b	Discussion of the relevant medical literature **with references**.	Discussion
	11c	The scientific rationale for any conclusions (including assessment of possible causes)	Discussion
	11d	The primary "take-away" lessons of this case report (without references) in a one paragraph conclusion	Conclusion
Patient Perspective	12	The patient should share their perspective in one to two paragraphs on the treatment(s) they received	Not reported
Informed Consent	13	Did the patient give informed consent? Please provide if requested	Yes ☒ No ☐ Consent section

Locations refer to manuscript sections in Final manuscript for production.
